# Nutzung von Krisendiensten vor Suizidversuchen: eine Datenerhebung in einem Krankenhaus der Maximalversorgung

**DOI:** 10.1007/s00115-022-01325-y

**Published:** 2022-05-30

**Authors:** Andreas Frank, Oscar Flissakowski, Florian Eyer, Peter Brieger, Johannes Hamann

**Affiliations:** 1 Klinik und Poliklinik für Psychiatrie und Psychotherapie, Klinikum rechts der Isar, Technische Universität München, Ismaninger Str. 22, 81675 München, Deutschland; 2 Fachklinik für Psychiatrie und Psychotherapie, Klinikum Fünfseenland Gauting, Gauting, Deutschland; 3 Abteilung für klinische Toxikologie & Giftnotruf München der II. Medizinischen Klinik, Klinikum rechts der Isar, Technische Universität München, München, Deutschland; 4 kbo-Isar-Amper-Klinikum Region München, München, Deutschland

## Hintergrund

In Deutschland sterben jährlich ca. 9000 Menschen an einem Suizid. Die Zahl der Suizidversuche liegt noch um ein Vielfaches darüber. Aktuelle Präventionsansätze zielen vor allem auf Früherkennung und Behandlung psychischer Erkrankungen ab, die als Hauptrisikofaktor für Suizide angesehen werden [1]. Einen weiteren wesentlichen Risikofaktor machen auch krisenhafte Zuspitzungen psychosozialer Belastungssituationen aus [2]. In diesem Zusammenhang kommt gemäß dem Bayerischen Psychisch-Kranken-Hilfegesetz 2018 den psychiatrischen Krisendiensten eine entscheidende Rolle zu, da diese in allen sieben bayerischen Regierungsbezirken flächendeckend 365 Tage im Jahr und rund um die Uhr mit geschultem Fachpersonal telefonisch (und teilweise aufsuchend) für Personen in psychischen Krisen und psychosozialen Notlagen zur Verfügung stehen [3]. In akuten Fällen kann der Krisendienst, aufgrund der guten Kenntnis der regionalen psychiatrischen Versorgungstrukturen, eine Vorstellung beim sozialpsychiatrischen Dienst, einer psychiatrischen Praxis oder auch die Einleitung einer Krisenintervention mit mobilen Teams vor Ort veranlassen [4]. Die Bedeutung der Krisendienste wurde auch im neuen Koalitionsvertrag der Bundesregierung mit dem Vorhaben eines flächendeckenden Ausbaus der psychiatrischen Notfall- und Krisenversorgung bestärkt [5]. Unklar ist jedoch, ob diese Einrichtungen Personen vor einem Suizidversuch überhaupt erreichen. Deshalb untersuchten wir in dieser Datenerhebung die Kenntnis und Nutzung von Krisendiensten bei Überlebenden von Suizidversuchen.

## Methode

Für die Datenerhebung wurden die psychiatrischen Konsile von Patienten, die sich im Zeitraum von 01.10.2019 bis 31.10.2020 aufgrund eines Suizidversuches in stationärer Behandlung im Klinikum rechts der Isar befanden, ausgewertet. Die Routinedatenerhebung der Konsile (d. h. eigene Angaben des Patienten, Vorgeschichte mit klinischen und soziodemographischen Daten, Art des Suizidversuches, psychiatrische Vorgeschichte und Diagnosen, psychosoziale Belastungsfaktoren, Suizidanamnese, Suchtanamnese) wurde insbesondere um Kenntnis und Nutzung von Krisendienstangeboten und ob ein Notfall- oder Krisenplan vorliegt ergänzt und so standardisiert, dass eine spätere Auswertung möglich wurde. Mittels SPSS Statistics for Windows, Version 26.0 (Armonk, NY, USA: IBM Corp) fand eine deskriptive Auswertung statt. Zudem wurden Personen, denen der Krisendienst bekannt war mittels T‑Tests und χ^2^-Tests mit denjenigen vergleichen, denen der Krisendienst nicht bekannt war. Die Datenerhebung wurde von der Ethikkommission der Technischen Universität München positiv begutachtet.

## Ergebnisse

Das Durchschnittsalter der 126 Patienten betrug 42,7 Jahre (von 15 bis 93 Jahren, SD 18,4) und 75 % (*n* = 95) waren Frauen. Als (psychiatrische) Hauptdiagosen der Patienten wurden vor allem affektive Erkrankungen (*n* = 76) gestellt. Mit 85,7 % (*n* = 108) der Patienten war die vorsätzliche Selbstvergiftung am häufigsten vertreten, gefolgt von einer Kombination verschiedener Suizidmethoden mit 7,1 % (*n* = 9). 48 % der Patienten befanden sich in regelmäßiger ambulant-psychiatrischer Behandlung. Bei 72 % bestanden psychosoziale Belastungsfaktoren, wobei am häufigsten „partnerschaftliche oder familiäre Konflikte“ angegeben wurden. Bei 29 % (*n* = 30 von 103) der Patienten wurde ein Krisen- und Notfallplan für Akutsituationen mit dem Behandler besprochen.

Bezüglich der Kenntnis des Krisendiensts gaben 55 % (*n* = 69 von 125) der Patienten an, dass ihnen dieser bekannt sei. Die Wahrscheinlichkeit den Krisendienst zu kennen, war dabei höher für Patienten, die schon in psychiatrischer Behandlung waren (*p* < 0,001), die einen Notfallplan hatten (*p* = 0,12), die bereits einen Suizidversuch in der Vorgeschichte hatten (*p* < 0,001) und die deutsche Muttersprachler waren (*p* < 0,001). Zudem gab es deutliche Unterschiede zwischen den Diagnosen (*p* = 0,001), wobei Patienten mit schizophrenen und Borderline-Erkrankungen häufiger den Krisendienst kannten, als Patienten mit affektiven Erkrankungen. Keine signifikanten Unterschiede fanden sich hinsichtlich Alter und Geschlecht der Patienten.

Nur 9 % (*n* = 11 von 121) der Betroffenen hatten im Vorfeld des Suizidversuches Kontakt zum Krisendienst aufgenommen. Als häufigsten Grund für eine fehlende Kontaktaufnahme gaben 42 % (*n* = 19 von 45) an, beim Krisendienst womöglich keine Hilfe zu erlangen. Als weitere Gründe für eine fehlenden Kontaktaufnahme bestanden bei 22 % (*n* = 10 von 45) akute Suizidgedanken oder eine Kurzschlussreaktion, bei 11 % (*n* = 5 von 45) bestanden Bagatellisierungstendenzen oder kein Behandlungswunsch, 6,6 % (*n* = 3 von 45) versuchten, anderweitig Hilfe zu bekommen, 11 % (*n* = 5 von 45) gaben sonstige, nicht kategorisierbare Gründe an und 6,6 % (*n* = 3 von 45) der Betroffenen befanden sich im stationären Setting.

## Diskussion

In der aktuellen Datenerhebung untersuchten wir die Kenntnis und Nutzung von Krisendiensten bei Überlebenden von Suizidversuchen in einem Universitätskrankenhaus. Bei den meisten Patienten wurde einerseits eine psychiatrische Diagnose gestellt, gleichzeitig lagen bei den meisten psychosoziale Krisen vor. Eigentlich sind Krisendienste ideale Anlaufstellen für Menschen in suizidalen Krisen, da sie sowohl bei psychischen Erkrankungen als auch bei psychosozialen Belastungssituationen Hilfe anbieten können [3]. Der Krisendienst war immerhin der Hälfte der Patienten bekannt, allerdings deuten die Ergebnisse an, dass der Krisendienst vor allem „psychiatrieerfahrenen“ Personen bekannt ist, also Menschen, die bereits in psychiatrischer Behandlung sind, schon Suizidversuche in der Vorgeschichte haben und unter schweren psychischen Erkrankungen leiden (z. B. Schizophrenie, Borderline-Persönlichkeitsstörung). Menschen, die ggf. erstmalig in eine psychosoziale Krise geraten und bei denen dann nach dem Suizidversuch eine affektive Erkrankung diagnostiziert wird, kennen den Krisendienst bisher kaum. In unserer Stichprobe nutzten allerdings weniger als 10 % der Betroffenen diese Unterstützungsmöglichkeit. Der häufigste Grund einer fehlenden Kontaktaufnahme trotz Bekanntheit war „beim Krisendienst womöglich keine Hilfe zu erlangen“. Als methodische Limitationen sind zu nennen, dass unsere Stichprobe nicht repräsentativ für alle Personen mit Suizidversuchen ist (hohe Anzahl an Selbstvergiftungen, durch internistisch-toxikologischen Schwerpunkt der Klinik) und es sich um Daten aus nur einer Klinik handelt. Auch geht aus unserer Untersuchung nicht hervor, welche Personen beim Krisendienst Hilfe erfuhren und somit womöglich Suizidversuche verhindert werden konnten. Zusammenfassend stellt sich dennoch die Frage, wie Krisendienste mit ihren Angeboten und vor allem ihrer Arbeitsweise bei Menschen in psychosozialen Notlagen besser angenommen und bekannter werden könnten. Alle verfügbaren Ansätze sollten dazu genutzt werden, u. a. regelmäßige Präsenz in der Medienberichterstattung, fortlaufend breite Verteilung von Infomaterialien, Informations- und Plakatkampagnen, wenn möglich in Kooperation mit anderen Versorgungspartnern oder namhaften öffentlichen Institutionen. Ärzte aller Fachdisziplinen wie auch Psychotherapeuten, psychosoziale Beratungsstellen, Rettungsdienste, Kliniknotaufnahmen und andere Multiplikatoren (z. B. Pfarrgemeinden) sollten selbst gute Kenntnis der Angebote von Krisendiensten haben und dies frühzeitig an Betroffene weitergeben. Das Ziel des bundesweiten Ausbaus von Krisendiensten im Koalitionsvertrag sollte zum Anlass genommen werden [5], zu analysieren und Strategien zu entwickeln, wie Krisendienste den Bedürfnissen von Menschen in suizidalen Krisen besser gerecht werden können.

## Fazit für die Praxis

- Psychiatrische Krisendienste sind etwa der Hälfte der Personen, die einen Suizidversuch begehen, bekannt. Nur etwa 10 % der Betroffenen haben vor dem Suizidversuch beim Krisendienst Hilfe gesucht.
- Krisendienste werden bislang überwiegend von Personen genutzt, die bereits Kontakt zum psychiatrischen Hilfesystem haben.
- Alle verfügbaren Ansätze sollten genutzt werden, um über Arbeitsweise und Angebote von Krisendiensten zu informieren – insbesondere auch bei Menschen, denen bislang das psychiatrische/psychosoziale Hilfesystem weniger bekannt ist.
